# Salicylic acid exerts effects on regulating flower bud differentiation and nutrient contents in red radish

**DOI:** 10.3389/fpls.2026.1914554

**Published:** 2026-09-01

**Authors:** Yan Zhang, Li Qiang, Dirong Chen, Yongyao Fu

**Affiliations:** 1 School of Advanced Agriculture and Bioengineering, Yangtze Normal University, Chongqing, China; 2 School of Life Science, Southwest University, Chongqing, China; 3 College of Biology and Food Engineering, Chongqing Three Gorges University, Chongqing, China

**Keywords:** flowering-related genes, physiological characteristics, red radish (*Raphanus sativus* L), response surface optimization, salicylic acid

## Abstract

Red radish (*Raphanus sativus* L. var. carminatus) is a characteristic economic crop in China, with flower bud differentiation efficiency directly determining its yield and quality. However, how salicylic acid (SA) affects this process and tuber quality remains unexplored. Here, we investigated different SA concentrations’ effects on endogenous hormones (GA_3_, IAA, CTK), flower bud differentiation, flowering, and pollen traits, alongside leaf soluble sugar, starch, and protein levels. We also applied response surface methodology (RSM) to build a regression model for optimizing SA application parameters, and conducted real-time PCR to detect the expression of flowering-related genes. SA and GA₃ most strongly affected apical buds (F = 45.2, p < 0.001). SA inhibited IAA in apical buds and seedlings (F = 35.8, p < 0.001), while functional leaf IAA peaked at 7% SA. Cytokinin peaked at 3% SA (F = 15.8, p < 0.001). Plant height, node number, leaf number and tubular flower number all reached their maximum values under 5% SA treatment. Starch content at the flowering stage increased significantly under 2% SA treatment, whereas the pre-anthesis protein accumulation reached the optimal level under 4% SA treatment. Pollen formation was significantly enhanced by 7% SA, boosting pollen quantity by 62.4%. The model optimization prediction indicated that the optimal nutrient contents could be obtained with the application of 5.09% SA for 24.82 days, verifying the parameters with <1% prediction error. Real-time PCR showed SA markedly downregulated flowering-related genes (RsGAIe, RsSLY1, RsUBC1h/g/b/i). These findings reveal that exogenous SA coordinately remodels endogenous GA₃-IAA-CTK hormone homeostasis to synchronously modulate flower-bud differentiation and leaf carbon-nitrogen accumulation in red radish (*Raphanus sativus* L. var. carminatus), and highlight a feasible strategy for manipulating flowering and improving economic traits via SA application. These findings provide a theoretical basis for shortening red radish’s flowering cycle and improving its economic traits.

## 1 Introduction

Red radish (*Raphanus sativus L.*), an important economic crop of the Brassicaceae family that is native to areas such as Fuling, Chongqing, China, has a long cultivation history and has been recognized as a Chinese National Geographical Indication Product (Lv et al., 2012; Tao et al., 2020). This variety is rich in natural carotenoid pigments, glucosinolates, vitamins, and mineral elements and is highly valuable for food processing and medicinal applications (Zhang et al., 2020; Chen et al., 2021). Plant pigments, anthocyanins are well known for their health benefits, such as antioxidant activity, anti-cancer effects, and enhancement of immunity (Butelli et al., 2008). Red-fleshed radish (*Raphanus sativus L*.), which has green or red skin and red flesh is a unique cultivar grown in China, where it is consumed in large quantities because it is rich in anthocyanins (Wang et al., 2020). With expanding market demand, optimizing cultivation techniques to increase the yield and quality of red radish has become a popular research topic.

Salicylic acid (SA), an endogenous phenolic compound with the chemical formula C7H603, modulates a wide range of plant growth and development processes by integrating into complex plant hormone signaling networks (Zhang et al., 2021). Accumulating evidence has demonstrated that SA possesses a remarkable capacity to alleviate plant stress damage (Henschel et al., 2022), and it also acts as a crucial regulator of plant flowering, physiological metabolism, and endogenous hormone homeostasis.

SA plays an essential regulatory role in the floral transition of plants. In *Arabidopsis thaliana*, SA bridges plant defense responses and reproductive development and modulates flowering time through the photoperiod and autonomous signaling pathways (Martinez et al., 2004). Moreover, SA cooperates with gibberellins and auxin to jointly regulate floral initiation (Luo and He, 2025). The mechanism of SA-induced flowering provides a valuable reference for elucidating how exogenous SA promotes floral bud differentiation in radish (Wada and Takeno, 2013). Numerous studies on plant physiological metabolism and nutrient accumulation have verified that SA regulates plant antioxidant systems and the accumulation of soluble sugars and proteins, thereby modulating multiple physiological processes in plants (Nouairi et al., 2024). In Brassicaceae species, exogenous SA exerts regulatory effects on photosynthesis, nitrogen assimilation, and substance accumulation. The SA-IAA signaling pathway has been proven to mediate carbohydrate and protein metabolism as well as starch and protein accumulation in plants (Meng et al., 2026). Furthermore, SA participates in the regulation of endogenous hormone balance and carbon-nitrogen metabolism. Notably, SA exhibits a concentration-dependent bidirectional effect on plant growth and flowering, following a typical pattern of low-concentration promotion and high-concentration inhibition (Pluhařová et al., 2019; Zhou et al., 2024). To date, direct research focusing on the regulatory mechanism of SA on floral bud differentiation in red radish remains limited. Nevertheless, the confirmed regulatory functions of SA in Cruciferae plants indicate that SA can modulate nutrient allocation and endogenous hormone balance to affect floral bud differentiation (Hao, 2019; Ding and Ding, 2020; Zhao et al., 2024). Specifically, SA facilitates the transport of photosynthates to flower buds, and synergistically promotes floral bud differentiation by increasing cytokinin levels and decreasing gibberellin contents in plants (Wang et al., 2024; Zhan et al., 2024).

On plant floral bud differentiation. how different SA concentrations modulate endogenous hormone levels and the accumulation of key nutrients (soluble sugars, sucrose, fructose, starch, and soluble proteins) in red radish leaves during floral bud differentiation remains unclear. Against this research background, the present study took red radish as the research material and further investigated the regulatory effects of varying SA concentrations on its flowering development and nutrient accumulation based on existing studies on SA-mediated nutrient regulation in plants. This study systematically analyzed the dynamic changes of endogenous hormones (GA3, IAA and CTK) in apical buds and mature leaves at different developmental stages during SA-regulated floral bud differentiation, as well as the comprehensive effects of SA on floral bud differentiation efficiency, flowering time, plant appearance quality and physiological activity. Additionally, response surface methodology (RSM) via Design-Expert software was applied to establish a predictive model, aiming to explore the effects of SA concentration on the accumulation of soluble sugars, sucrose, fructose, starch and soluble proteins, analyze the interactive relationships among regulatory factors underlying SA concentration adaptation mechanisms, and screen the optimal SA concentration for red radish cultivation. Furthermore, we further identified and analyzed genes associated with flowering in red-fleshed radish, and verified their expression levels using quantitative real-time polymerase chain reaction (qRT-PCR).The ultimate objectives of this study are to shorten the flowering period, improve yield, and promote the full development of fleshy roots in red radish. Collectively, the findings of this study can provide a solid theoretical basis for clarifying the SA-mediated physiological regulation mechanism in red radish, as well as support the exogenous application of SA to regulate flowering and improve crop quality, thereby facilitating the high-quality and high-yield cultivation of red radish in China.

## 2 Materials and methods

### 2.1 Experimental materials

The test material was Tongguan red radish (variety registration number: GS09001-2020), provided by Yunfeng Seedling Co., Ltd. Seedlings that were healthy and uniform in shape were selected and planted in a substrate containing Hoagland nutrient solution (pH 6.5 ± 0.2). The cultivation conditions included a light/dark cycle of 14 h/10 h, a temperature of 25 ± 2 °C/18 ± 2 °C, and a relative humidity of 65 ± 5%.

### 2.2 Single-factor experimental design

#### 2.2.1 Sample selection and concentration gradient screening

After the visual statistics for each group of red radishes were averaged, seedlings whose growth characteristics, such as plant height, stem thickness, number of nodes, and leaf number, were uniform were selected. Salicylic acid (SA) was dissolved with anhydrous ethanol (final ethanol concentration in solution< 0.5%, v/v), and 0.05% (v/v) Tween-20 was added as a spreading agent to prepare serial solutions with different mass fractions, which were freshly prepared prior to each application. Foliar spraying was conducted between 9:00 and 10:00 every 3 days, with a uniform volume of 8 mL solution per plant. The apical buds and leaves were sprayed evenly until the liquid film formed on the surface without dripping off. Preliminary experiments revealed that all plants died when sprayed with SA solutions at mass fractions exceeding 10% (Zhu et al., 2022). A concentration gradient ranging from 1% to 10% was set for preliminary screening, and plant survival rate exceeded 85% under treatments of 1%–8% SA. Based on phytotoxic symptoms and plant growth status, six concentrations (2%, 3%, 4%, 5%, 6%, 7%) were selected for the formal experiment, with clean water serving as the control. Each treatment contained five biological replicates. Continuous spraying lasted for 20–30 days to cover the critical period of flower bud differentiation of *Raphanus sativus L.* Tonguan Hongxin. Endogenous hormones and multiple physiological parameters of apical buds, young leaves and functional leaves were determined at the flower bud differentiation stage (20–30 days after treatment) and successive phases of floral induction (15–20 days before anthesis, 20–30 days during anthesis, and 30–35 days after anthesis). Cultivation conditions were maintained consistently throughout the experiment.

#### 2.2.2 Dynamic changes in endogenous hormone contents in red radish during flower bud differentiation

On the basis of flower bud anatomical observations, apical buds, apical buds whose small leaves were removed, and mature leaves corresponding to these periods were selected, rapidly frozen in liquid nitrogen, and stored at -80 °C. GA_3_, IAA, and CTK contents were measured using HPLC–MS/MS (Agilent 1260-6460) following the methods of Chen et al. (2019).

#### 2.2.3 Effects of SA on the bud differentiation and flowering of radish

During the experimental period, the apical bud differentiation process was observed daily at a fixed time (9:00 a.m.) at a 24-hour observation interval using a stereomicroscope (OlympusSZX16, 10×magnification), and the developmental stages were divided according to the standards of Zhang et al. (2020).

Observation and evaluation of plant appearance quality: The flowering process after seven different SA concentration treatments was statistically analysed, with a focus on recording the full bloom period. Stem diameter was measured with a Vernier calliper (0.01 mm), plant height and internode length were measured with a ruler, and fully expanded leaves and node numbers were counted manually. Flower neck, flower diameter, flower appearance (number of ligulate flowers and number of tubular flowers) and other data were used to evaluate the appearance quality of plants (Wang et al., 2023; Suy and Pan, 2024).

#### 2.2.4 Measurement of physiological indicators

##### 2.2.4.1 Carbohydrates

To determine the soluble sugar content, 0.2 g of cochineal radish leaves was ground in a small amount of quartz sand and 2 mL of 80% ethanol until a homogenate was obtained. The sample was transferred to a 10 mL centrifuge tube, and then, the mortar was washed with 2 mL of 80% ethanol, which was also transferred to the 10 mL centrifuge tube, followed by incubation in an 80 °C water bath for 30 minutes. The centrifuge tube was removed and rinsed under running water to cool to room temperature, after which it was centrifuged at 8000 rpm for 20 minutes. The supernatant was transferred to a 10 mL centrifuge tube, precipitated and extracted again with 2 mL of 80% ethanol; the above steps were repeated, after which the supernatants were combined. To the obtained supernatant, 10 mg of activated charcoal was added, the sample was decolorized in an 80 °C water bath for 30 minutes, and the extract was filtered through filter paper to obtain a pale yellow extract. The volume was adjusted to 25 mL with 80% ethanol, and the sample was stored at 4 °C.In a precooled test tube, 0.2 mL of ethanol extract and 1.8 mL of H_2_O were added, 5 mL of anthrone reagent was added, and the tube was covered with a glass stopper. The sample was gently shaken left and right for 30 s, incubated in a 100 °C water bath for 10 minutes, and then rinsed under running water to cool to room temperature. The absorbance was measured at a wavelength of 620 nm.

To determine the sucrose content, 0.08 ml of total sugar extract, 0.32 mL of H_2_O, and 200 µL of 2 M NaOH solution were added to a test tube, which was boiled at 100 °C for 5 minutes and then cooled, after which 2.8 mL of 30% HCl was added to neutralize the previously added NaOH solution. At this point, the sucrose had been hydrolysed into fructose; therefore, the fructose determination method was used to determine the sucrose content.

To determine the starch content, 2 mL of distilled water was added to the total sugar extract precipitate, followed by gelatinization in a boiling water bath for 15 minutes. After the samples were allowed to cool to room temperature, 2 mL of 9.2 N perchloric acid solution was added, and the mixture was stirred for 15 minutes. Then, 4 mL of distilled water was added, and the solution was mixed thoroughly. The mixture was centrifuged at 10, 000 r/min for 10 minutes, and the supernatant was collected and transferred to a 50 mL volumetric flask. The remaining precipitate was then washed with 2 mL of 4.6 N perchloric acid solution for 15 minutes and centrifuged again at 10, 000 r/min for 10 minutes, after which the supernatant was combined into a 50 mL volumetric flask, and the volume was increased. One millilitre of the extract, 1 mL of distilled water and 5 mL of anthrone–sulphuric acid reagent were added sequentially, and the colour was developed in a boiling water bath for 10 minutes. The sample was cooled to room temperature, and the absorbance was measured at 620 nm to determine the starch content.

##### 2.2.4.2 Protein: coomassie brilliant blue G-250 method (595 nm)

The protein content was determined using the Coomassie Brilliant Blue G-250 method. A total of 0.2 g of plant sample was weighed into a mortar, followed by the addition of 2 mL of distilled water, and the sample was ground thoroughly until a uniform paste formed. Then, the paste was transferred to a centrifuge tube, the mortar was washed twice with 4 mL of distilled water, and the washings were collected into the same centrifuge tube. The mixture was allowed to stand at room temperature for 0.5–1 hour and was then centrifuged at 10, 000 r/min for 6 minutes. After centrifugation, the supernatant was transferred to a 10 mL graduated tube and brought to 10 mL with distilled water to prepare the sample solution to be tested. This solution (0.1 mL) was added to a stoppered test tube, after which 5 mL of Coomassie Brilliant Blue solution was added. The contents were mixed quickly and allowed to stand at room temperature for 2 minutes, after which the absorbance was measured at 595 nm to determine the protein content.

#### 2.2.5 Reproductive characteristic analysis.

##### 2.2.5.1 Pollen quantity statistics

Anthers at full bloom were removed, dissociated with pectinase (2.5%) and counted with a haemocytometer; observations were repeated five times per sample. The formula was as follows (Wang et al., 2023; Wang et al., 2023):

(1) Pollen quantity(grains/anthers)=(N×D×V)/n,

where N is the mean count, D is the dilution factor, V is the total volume, and n is the number of anthers.

##### 2.2.5.2 Pollen viability

The iodine–potassium iodide (I_2_-KI) staining solution was prepared according to the method described by Jia and Liu (2007). Briefly, 2 g of potassium iodide was dissolved in 7 mL of distilled water, followed by the addition of 1 g of iodine crystals. After complete dissolution, the solution was diluted to a final volume of 100 mL and stored in a brown reagent bottle away from light. For pollen viability detection, several mature anthers were placed on a clean glass slide and gently crushed with an inoculating needle to release pollen grains. One drop of I_2_-KI staining solution was added to fully cover the pollen sample, and the slide was incubated at room temperature for 10 min prior to microscopic observation. Under the microscope, pollen grains stained dark blue were defined as viable, while those appearing yellowish-brown were considered inactive and non-viable. Each sample was examined in three independent experimental replicates, with three random microscopic fields selected per replicate (no fewer than 20 pollen grains per field). The pollen viability percentage was calculated according to the corresponding formula (Huang etal., 2015).

(2) Viability rate=stained pollen count/total pollen count×100%

### 2.3 Response surface experimental design

On the basis of the results of a single-factor experiment, a Box–Behnken design (Design Expert 12.0) was used, with SA concentration (A) and treatment period (B) as independent variables, to establish a quadratic regression model:

(3) $$\text{Y}=\beta_{0}+\beta_{1}\text{A}+\beta_{2}\text{B}+\beta_{11}{\text{A}}^{2}+\beta_{22}{\text{B}}^{2}+\beta_{12}\text{AB.}$$

With the response variables being protein, total sugar, and starch contents. The coded levels of the factors are shown in **Tables 1**, **2**.

**Table 1:** Two-factor three-level response surface analysis experimental design table.

Factor	Symbol	Unit	Coding level		
			-1	0	+1
SA Concentration	A	%	3	5	7
Treatment Time	B	d	15 (preflowering)	25 (flowering)	35 (postflowering)

**Table 2:** Response surface optimization experimental design (Box–Behnken Design).

Number	A: Concentration (%)	Encoding value A	B: Periods (d)	Encoding value B	Protein (mg)	Sugar (mg)	Starch (mg)
1	3	-1	15	-1	40.43	48.96	51.48
2	7	+1	15	-1	40.15	47.57	56.16
3	3	-1	35	+1	39.52	44.08	57.72
4	7	+1	35	+1	38.68	49.48	58.50
5	3	-1	25	0	41.73	49.48	59.28
6	7	+1	25	0	42.43	51.23	60.84
7	5	0	15	-1	44.99	53.98	62.40
8	5	0	35	+1	42.91	52.77	63.96
9	5	0	25	0	47.41	55.21	68.64
10	5	0	25	0	48.32	55.68	67.08
11	5	0	25	0	47.72	57.96	67.08
12	5	0	25	0	47.72	57.77	67.86
13	5	0	25	0	48.53	56.72	68.64

### 2.4 Spatiotemporal expression analysis of key enzyme genes

Arabidopsis flowering-related genes were downloaded from the TAIR10 database, and radish genome information was acquired from the website http://radish-genome.org. BLASTn alignment was carried out between Arabidopsis flowering genes and the radish reference genome (Pastermak et al., 2019). In the similarity-based approach, BLASTP searches were conducted against R. sativus protein sequences using the following conditions: E-value<1e-20, identity >50%, coverage >60%, and match length > 60 amino acids.

Total RNA was extracted from leaves of three independent batches treated with different SA concentrations (5% and 6%) for varying durations (0, 12, 24 h). Thereafter, cDNA was synthesized by reverse transcription. Using cDNA as templates, real-time quantitative PCR (qRT–PCR) was employed to analyse the relative expression levels of key enzyme genes in *Raphanus sativus L*. RNA integrity and reverse transcription procedures were performed following standard protocols.

The qRT–PCR reaction system was programmed as follows: initial preheating at 94 °C for 3 min; 35 cycles of denaturation at 94 °C for 30 s, annealing at 60 °C for 30 s, and extension at 72 °C for 30 s; and a final extension at 72 °C for 7 min. Three technical replicates and one negative control were arranged for all samples. To validate the specificity of qRT–PCR amplification, melting curve analysis was conducted post-amplification. The thermal program was set to gradually increase the temperature from 60 to 95 °C, with fluorescence intensity detected at 0.5 °C increments every 5 s for continuous signal recording.

Actin was used as the internal reference gene. Primer sequences for target genes are presented in **Table 3**. Each sample included three biological replicates. All quantitative data were analysed using the 2^–^ΔΔCt method (Livak method).

**Table 3:** Primers for real-time quantitative PCR.

Primer name	Primer sequences (5 ′-3 ′)
Actin-F	GTACGACAGGTATCGTTATGGA
Actin-R	ACCAGCAAGATCCAACCTCAA
dRsGA1e-F	ATGAAGAGAGATCTCCATC
dRsGA1e-R	GCTCCAGCTTCAACGCGACC
dRsSLY1-F	ATGAAACGCAGTGCTTC
dRsSLY1-R	CAGCTCCCACAGACGCTCG
dRsUBC1g-F	ATGTCGACTCCAGCGAGGAAG
dRsUBC1g-R	CCGTGAAACAAACCGAACAG
dRsLBC1h-F	ATGGATACAAATACATCTGG
dRsLBC1h-F	GCCTCTTTCTCCAACTTGG
dRsUBC1b-F	ATGGCATCGCAAGCTAGCC
dRsUBC1b-R	CCTCCTTCATAGAGAGTATC
dRsUBC1i-F	ATGTCGACACCAGCAAGGAAG
dRsUBC1i-R	CATCCGTGACACAAAACGAAC

### 2.5 Data statistics

One-way analysis of variance (ANOVA) was performed using SPSS 25.0, with Duncan’s multiple comparison (α=0.05). The response surface data underwent quadratic polynomial fitting, with model significance assessed via the coefficient of determination (R^2^) and F test. The data are presented as the mean ± SE, and the figures were generated using Origin 2022b.

## 3 Results

### 3.1 Effects of SA treatment on changes in endogenous hormone levels during flower bud differentiation

The data in **Table 4** reveal that the growth stage significantly dominated the GA_3_ dynamics (F value > 500), with the highest content observed during the seedling stage and the lowest during the mature stage. The interaction between SA and growth stage was also significant, indicating that SA regulation depends on developmental stage.SA represses GA20ox and GID1 accumulation to stabilize DELLA proteins, inhibiting FT/SOC1 transcription and GA-mediated floral bud differentiation (Yu et al., 2022).

**Table 4:** Two-way ANOVA results for SA concentration and growth stage effects on GA_3_ content.

Source	Apical buds GA_3_	Young leaves GA_3_	Functional leaves GA_3_
	F (df)	F (df)	F (df)
SA Concentration	45.2*** (7, 48)	32.8*** (7, 48)	18.3*** (7, 48)
Growth Stage	1285.1*** (2, 48)	878.4*** (2, 48)	579.6*** (2, 48)
SA × Concentration	6.6*** (14, 48)	5.4*** (14, 48)	3.3** (14, 48)

F-Number: The brackets contain degrees of freedom (between-group degrees of freedom, error degrees of freedom), ***p< 0.001, **p< 0.01, *p< 0.05, Values without marks are considered not significant.

With respect to IAA, the data in **Table 5** reveal that elevated SA concentrations significantly suppressed IAA accumulation in both apical buds and young leaves. While functional leaves exhibited a significant but weaker effect, IAA levels in functional leaves were lowest with 7% SA. Interaction effects (SA×growth stage) were extremely significant for apical buds (F = 8.2, p<0.001), with the inhibitory effect of SA on IAA being strongest during the seedling stage. The interaction for young leaves was significant (F = 6.9, p<0.001), with SA exhibiting more pronounced IAA inhibition in young leaves during the germination stage; the interaction for functional leaves was significant (F = 4.1, p<0.01), and SA inhibition of IAA in functional leaves was weakest during the mature stage.SA inhibits YUC and PIN to lower SAM IAA, dampening ARF5-activated LFY, consistent with our SA-induced IAA reduction (Lin et al., 2018;Lin et al., 2019);.

**Table 5:** Two-way ANOVA results for SA concentration and growth stage effects on IAA content.

Source	Apical buds IAA	Young leaves IAA	Functional leaves IAA
	F (df)	F (df)	F (df)
SA Concentration	35.8*** (6, 42)	28.4*** (6, 42)	12.6*** (6, 42)
Growth Stage	620.5*** (2, 42)	480.3*** (2, 42)	85.2*** (2, 42)
SA × Concentration	8.2*** (12, 42)	6.9*** (12, 42)	4.1** (12, 42)

F-Number: The brackets contain degrees of freedom (between-group degrees of freedom, error degrees of freedom), ***p< 0.001, **p< 0.01, *p< 0.05, Values without marks are considered not significant.

As shown in **Table 6**, the SA concentration had extremely significant effects in apical buds (F = 22.6, p<0.001): low SA concentrations (2%-3%) promoted CTK accumulation, whereas high SA concentrations (5%-7%) inhibited it. In young leaves, the effect was extremely significant (F = 18.3, p<0.001), with CTK peaking with 3% SA and significantly decreasing with 7% SA. In functional leaves, the effect was significant (F = 15.8, p<0.001). The interaction (SA × growth stage) showed that the interaction in apical buds was significant (F = 5.4, p<0.001), with 3% SA having the strongest promoting effect on CTK during the germination stage. The interaction in young leaves was extremely significant (F = 7.1, p<0.001), with 3% SA significantly increasing CTK during the seedling stage. The interaction in functional leaves was significant (F = 4.2, p<0.01), with 3% SA showing the most pronounced effect during the seedling stage.SA exerts dual concentration-dependent control over CTK: low SA upregulates IPT/WUS to boost CTK and floral primordia, while high SA activates CKX to degrade CTK and retard bud differentiation (Werner T et al., 2003), This mechanism well explains our observation that 3% SA significantly increases CTK content while 7% SA drastically reduces CTK abundance.

**Table 6:** Two-way ANOVA results for SA concentration and growth stage effects on CTK content.

Source	Apical buds CTK	Young leaves CTK	Functional leaves CTK
	F (df)	F (df)	F (df)
SA Concentration	22.6*** (6, 42)	18.3*** (6, 42)	15.8*** (6, 42)
Growth Stage	480.2*** (2, 42)	620.5*** (2, 42)	350.4*** (2, 42)
SA × Concentration	5.4*** (12, 42)	7.1*** (12, 42)	4.2** (12, 42)

F-Number: The brackets contain degrees of freedom (between-group degrees of freedom, error degrees of freedom), ***p< 0.001, **p< 0.01, *p< 0.05, Values without marks are considered not significant.

SA fails to directly bind FT, LFY and FLC promoters; it reshapes GA_3_-IAA-CTK homeostasis and coordinates multiple flowering pathways to modulate floral differentiation indirectly (Martínez et al., 2004). Our hormonal data merely show correlations without molecular proof of direct SA causality.

### 3.2 Effects of SA on flower bud differentiation and flowering in red radish

**Figure 1:**
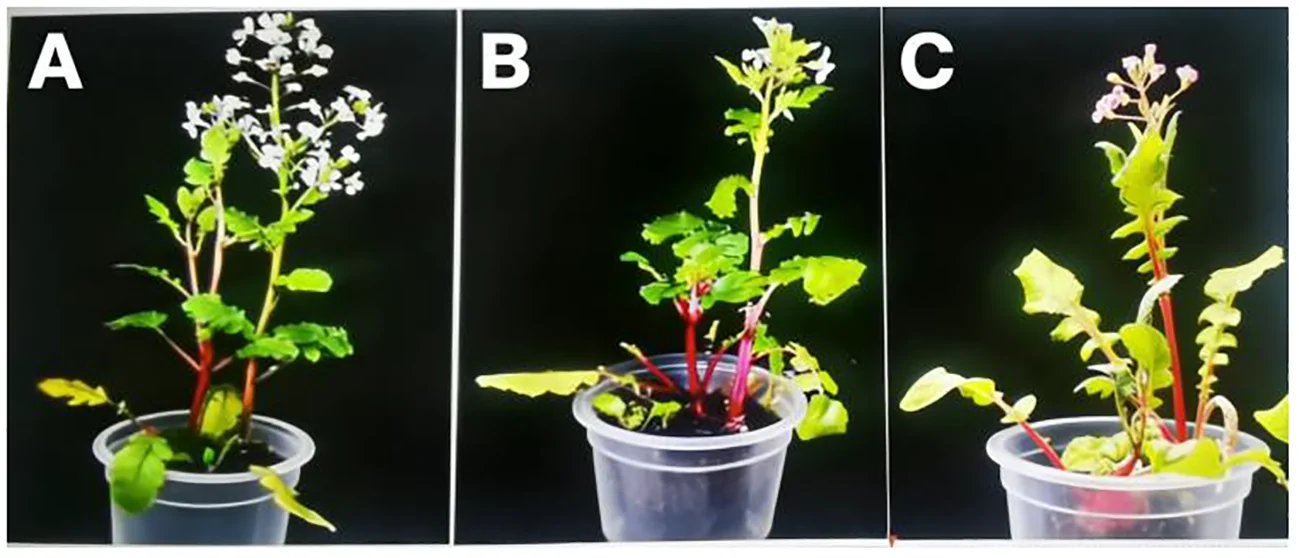
Appearance of flowering in carmine radish (*Raphanus sativus L*. var. niger Makino) as affected by salicylic acid (SA) treatment. **(A, B)** Effects of 5% and 7% salicylic acid on the floweringof the red radish *(Raphanus sativus L.)* respectively; **(C)** CK.

As shown in **Table 7**; **Figure 1**, compared with the other treatments, 5% SA significantly increased the plant height, node number, leaf number, and number of ligulate and tubular flowers in all the groups. These findings indicate that 5% SA significantly promotes both vegetative growth (stem elongation and increased branching) and reproductive growth (flower organ development); for instance, as expressed in Equation 1, the number of ligulate and tubular flowers reached 37, which was 8 times that of the CK group, Indicate that the strong promotion effect of 5% SA on floral organs. Compared with that in the other groups, the stem diameter (1.67 mm) in the 2% SA group was significantly greater, possibly because of SA-induced cell enlargement; however, plant height was lower (14.87 cm), indicating that low-concentration SA promotes stem thickening rather than elongation. No significant effects or outliers were detected for flower diameter. The mean flower diameter ranged from 0.72 to 0.88 across all concentrations, with ANOVA revealing no significant difference (p=0.89), indicating a negligible influence of SA concentration on flower diameter.SA exerts concentration-dependent dual regulation on plant vegetative and reproductive development. Low-dose SA (2%) prioritizes stem thickening to strengthen plant mechanical support, while moderate 5% SA balances vegetative biomass accumulation and floral organ differentiation to greatly boost flower yield. In contrast, SA application does not alter flower radial growth, suggesting that SA mainly modulates cell division rather than cell expansion in floral organs. This concentration-specific growth regulation of SA provides a feasible exogenous regulation strategy to coordinate plant vegetative and reproductive growth.

**Table 7:** One-way ANOVA and multiple comparisons (LSD test, α=0.05) for SA treatment effects on morphological traits in the radish (*Raphanus Sativus L*. var. Radiculus Pers.).

Indicator	F Number	P Number	Significance	Multiple comparison results (mean marking)
Plant Height	18.92	<0.001*	significant	SA5 (A) > SA7 (B) > SA3 (C), CK (C) > SA6 (D), SA4 (D) > SA2 (E)
Stem Diameter	5.83	0.001	significant	SA2 (A) > SA5 (B) > SA3 (C) > SA4 (D), SA7 (D), SA6 (D) > CK (E)
Node Number	36.54	<0.001*	significant	SA5 (A) > SA7 (B) > SA4 (C), SA6 (C), SA3 (C) > SA2 (D), CK (D)
Leaf Number	7.62	<0.001*	significant	SA5 (A) > SA2 (B), SA4 (B), SA7 (B) > SA3 (C) > CK (D), SA6 (D)
Flower Diameter	0.34	0.89	significant	no significant differences
Ray Floret/ Tube Floret Number	16.31	<0.001*	significant	SA5 (A) > SA7 (B), SA2 (B) > SA3 (C) > CK (D), SA4 (D), SA6 (D)

F Number and P Number: *indicates p<0.05 (significant), ns indicates not significant, different capital letters in the same column indicate significant between-group differences (P<0.05, LSD test), and marking of means are ordered from highest to lowest. Differences within the same letter group are not significant. The values in parentheses represent the means of each treatment group (e.g., SA5 = 39.53), and CK refers to the control group.

### 3.3 Effect of SA on pollen activity in the red radish

As shown in **Table 8**, the pollen count calculated via Equation 1 in the 7% SA group (35, 400 grains) was extremely significantly greater than that in all the other treatment groups (labelled A), and that in the 5% SA group (30, 400 grains) was significantly greater than that in the CK and 6% SA groups but lower than that in the 7% SA group (labelled B). however, no significant differences were detected among the 2%, 3%, and 4% treatment groups (labelled as BC), although all the values were significantly greater than those in the CK and 6% SA groups. The control group (CK) presented the lowest pollen count, which was significantly lower than that in all the other treatment groups except the 6% SA group.Appropriate exogenous SA supply can facilitate anther development and pollen formation, while excessively high SA concentration (6%) fails to promote pollen production. Moderate SA application (2%–5%) improves male reproductive capacity, which is conducive to pollen maturation, pollination and subsequent fruit setting during reproductive development.

**Table 8:** Analysis of the effects of treatment concentration on pollen quantity (one-way ANOVA with Tukey’s HSD test).

Treatment concentration (%)	Pollen grains number (grains)	Significant difference^a^	Result of analysis of variance (F number and P number)
7	35, 400 ± 1200	A	
5	30, 400 ± 980	B	
3	29, 200 ± 860	BC	F_(6, 35)_ = 3.62
2	29, 000 ± 750	BC	p = 0.009
4	28, 200 ± 920	BC	
6	25, 200 ± 680	C	
CK	21, 800 ± 550	D	

Different letters indicate significant between-group differences (Tukey HSD test, α = 0.05). The analysis of variance showed that different treatments had a significant effect on pollen quantity (p< 0.01), and between-group variation accounts for 37.9% of the total variation.

Statistical calculations were performed based on Equation 2. The ANOVA results yielded an F value of 5.03 (P<0.01), indicating extremely significant differences in pollen activity among treatments (α=0.01). As shown in **Table 9**, compared with the other groups, the 5% SA group (47.73%) exhibited extremely significant pollen activity (P<0.01), with the strongest promotion effect. Compared with the control group (CK), the 2%, 3%, and 7% SA groups presented significantly greater activity, but the activity did not significantly differ (all belonging to Group B) however, the activity in the 4% SA and 6% SA groups did not significantly differ from that in the control group (P>0.05), although that in the 4% SA group was marginally greater. Additionally, 2% SA, 3% SA, and 7% SA (low to medium concentrations) promoted activity, with activity 31–80% higher than that in the CK group. The activity in the 6% SA and 4% SA groups (higher concentrations) was close to or slightly above that in the CK group but did not reach significance, potentially indicating a “concentration threshold effect”.

**Table 9:** Multiple comparisons (Duncan’s Method, α=0.05).

Treatment concentration (%)	Mean (%)	Group (significantly different groups)
5	47.73	A
3	34.21	B
2	30.97	B
7	29.03	B
6	26.02	B
4	21.65	B
CK	19.01	C

F Number = 5.03, P<0.01.

### 3.4 Effects of SA treatment on red radish nutritional content

#### 3.4.1 Dynamic changes in soluble sugar content

**Figure 2:**
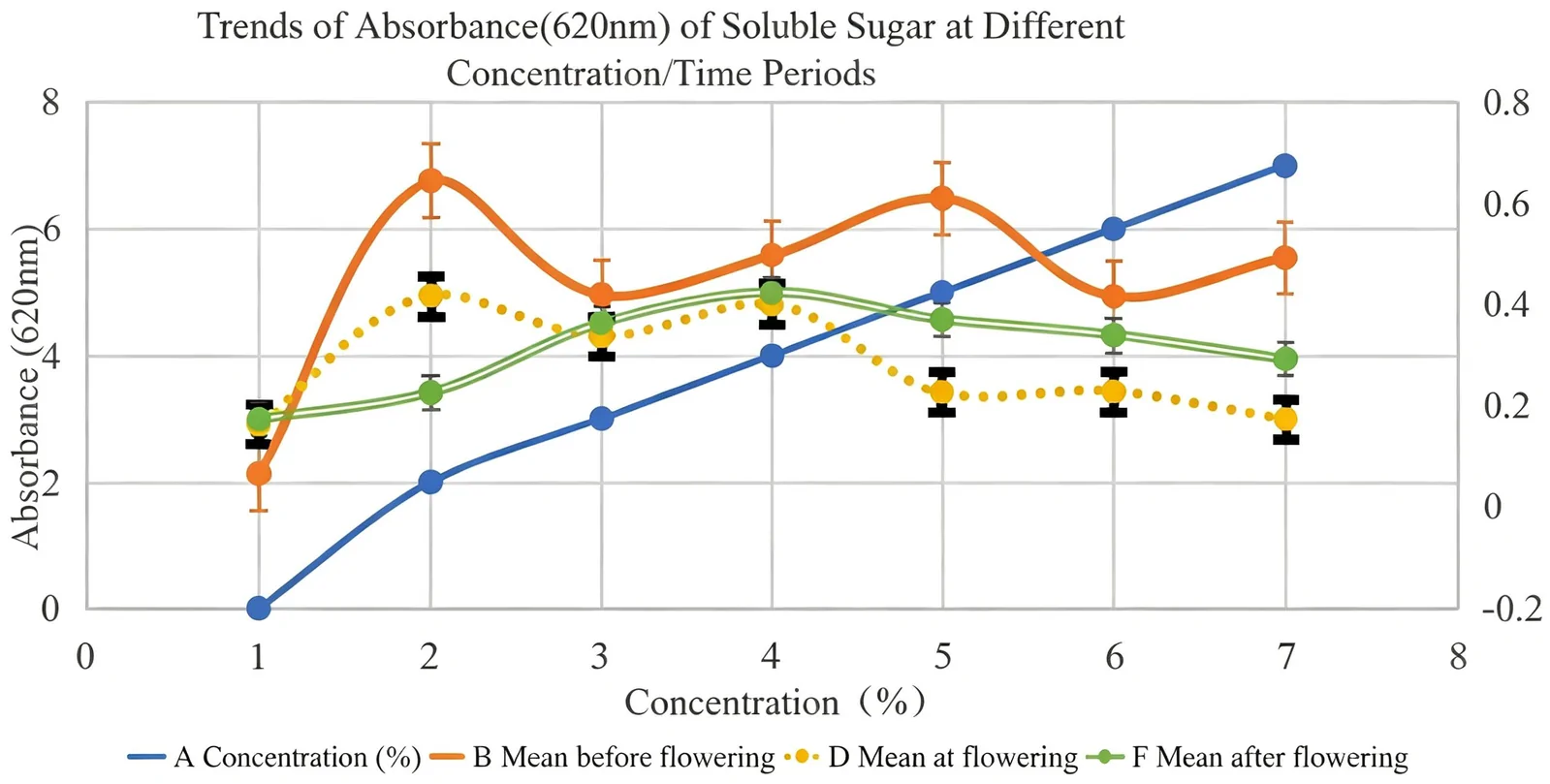
Absorbance (620 nm) of soluble sugar as a function of different concentrations/time periods.

The data in **Figure 2** show that the soluble sugar content in the CK group increased gradually across the growth stages (preflowering → postflowering: 0.070 → 0.175), with low change rates (preflowering → flowering +136%, flowering → postflowering +6%). With 2% SA, the preflowering absorbance peaked significantly higher than that with the CK (p<0.05), followed by a sharp decline from flowering to postflowering (-45% change rate). SA at 5% resulted in peak preflowering absorbance, a significant decrease during flowering (-62%), and a rapid rebound postflowering (+62%) (as shown in Table 2.6). SA at 7% resulted in a decrease from preflowering to flowering (-45%) and a recovery postflowering (+35%) but weaker stability. The preflowering absorbance was moderate with 3%, 4%, and 6% SA, with inconsistent treatment effects. SA at 4% yielded stable performance (standard deviation<0.1 across all stages). Data related to the dynamic changes in soluble sugar content are shown in **Supplementary Tables A1**, **A2**.

Correlation analysis with flowering indicators revealed synergistic advantages with 5% SA. The re-accumulation of postflowering sugars may prolong leaf functional periods, increasing seed plumpness. SA at 2% posed potential risks, with the highest preflowering content but the lowest postflowering content.

#### 3.4.2 Dynamic changes in sucrose content

**Figure 3:**
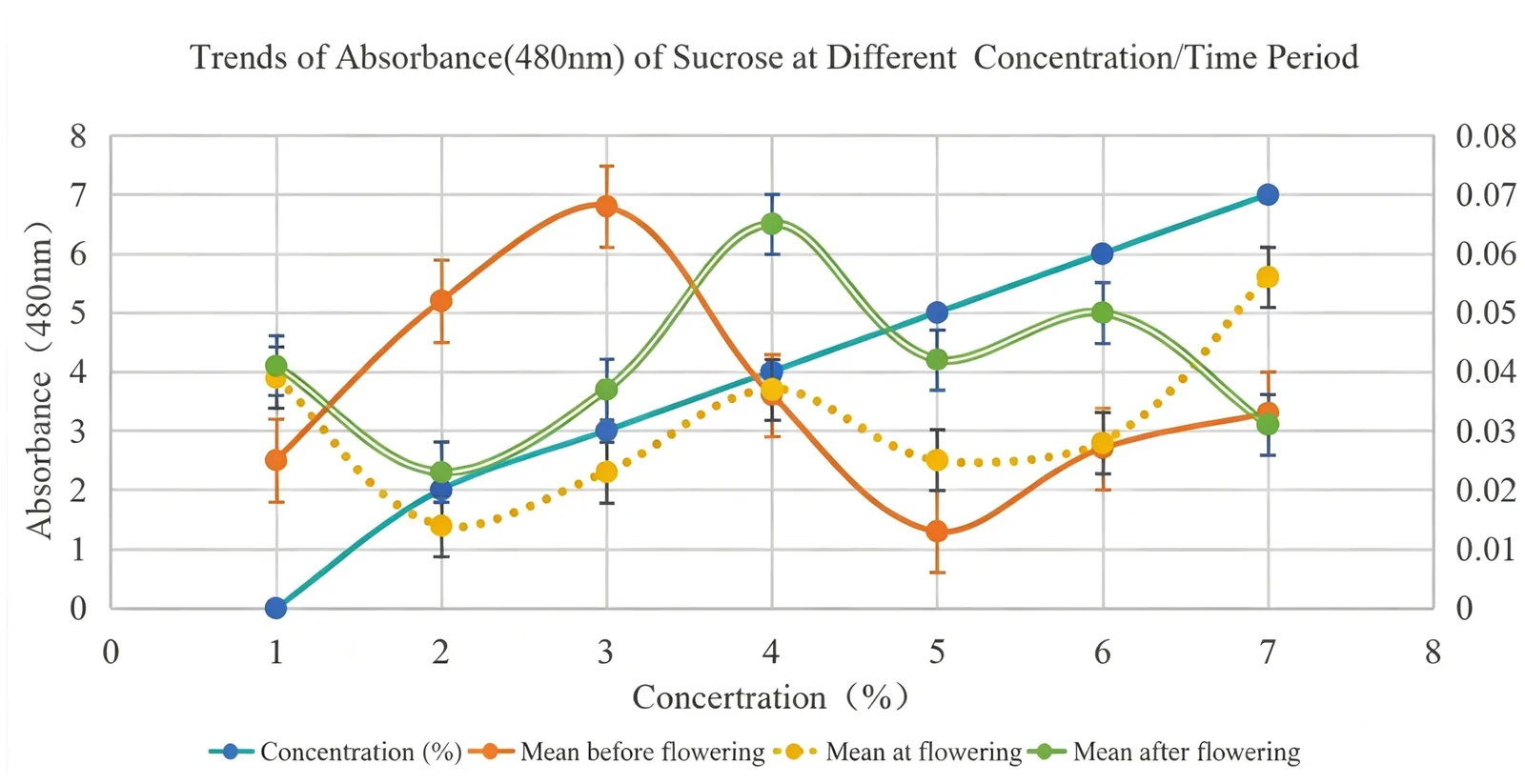
Absorbance (480 nm) of Sucrose as a function of different concentrations/time periods.

As shown in **Figure 3**, during the preflowering stage (initial sucrose accumulation), 3% SA significantly promoted sucrose accumulation, with a mean preflowering absorbance of 0.068 (CK = 0.025), whereas 5% SA inhibited sucrose accumulation, yielding a mean absorbance of only 0.013. During flowering (rapid sucrose consumption), sucrose concentrations generally decreased. Except for the CK group, in which the absorbance increased from 0.025 to 0.039, 2%, 3%, and 5% SA significantly decreased the sucrose content (50%-70% reduction). With 4% SA, the sucrose content increased from 0.037 during flowering to 0.065 postflowering (↑76%), and with 6% SA, the absorbance was 0.050 (↑79% compared with flowering), but with minimal data fluctuation (SD = 0.009) and good stability. Compared with the CK treatment (0.025), 5% SA resulted in continuous inhibition, with the postflowering absorbance (0.042) remaining lower. Data related to the dynamic changes in sucrose content are shown in **Supplementary Tables A3**, **A4**.

#### 3.4.3 Dynamic changes in starch content

**Figure 4:**
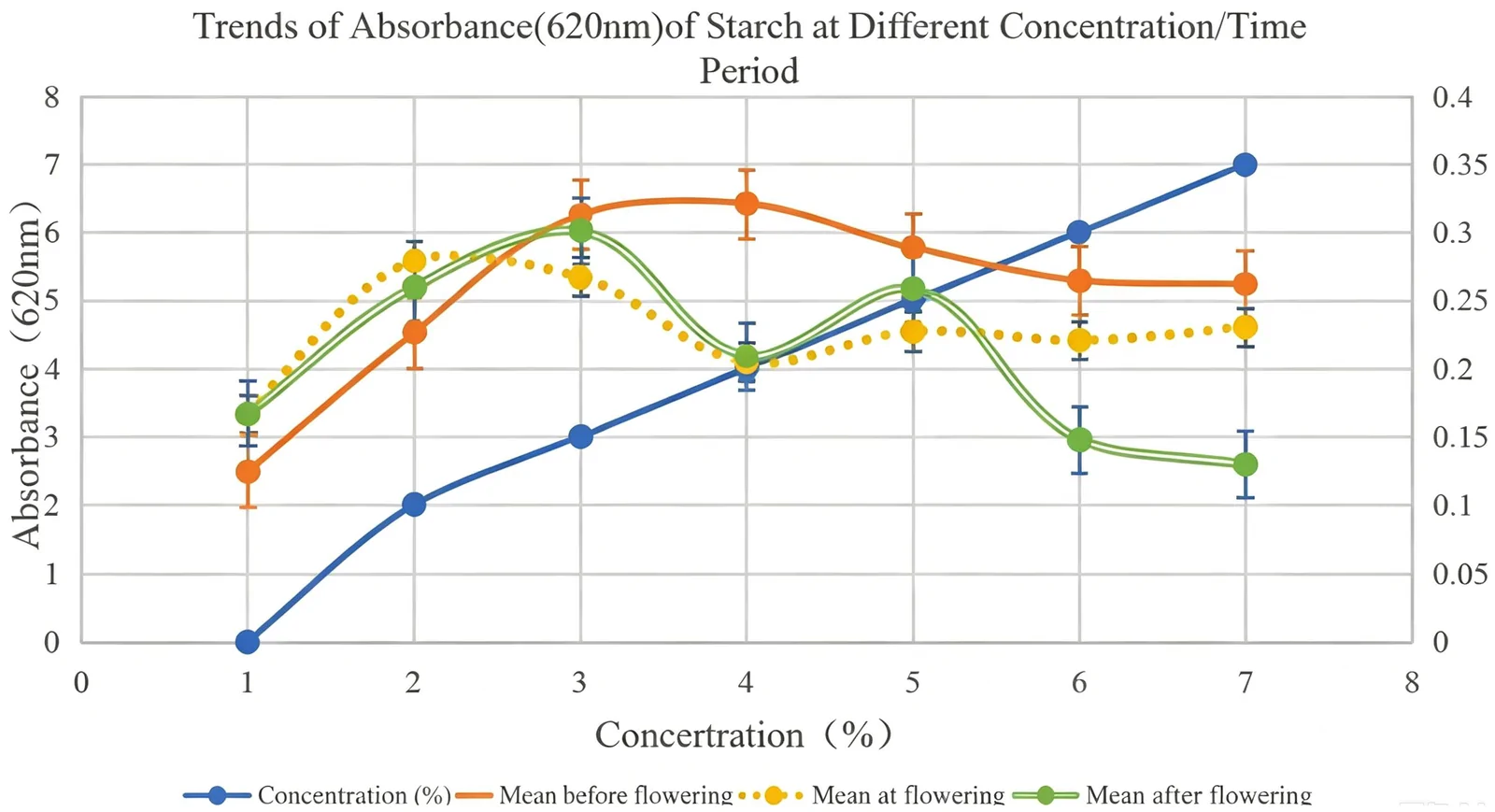
Absorbance (620 nm) of starch as a function of different concentrations/time periods.

Different SA concentrations significantly influenced the starch content (p<0.05), with 2%, 3%, 5%, and 7% SA having particularly pronounced regulatory effects. No significant interactions were detected, and the regulatory effects of SA concentration on starch did not significantly differ across different periods. The results of the analysis shown in **Figure 4** revealed that the transport characteristics from preflowering to flowering were characterized by CK, and compared with the preflowering conditions, 6% and 7% SA resulted in a significant increase in starch absorbance during flowering. With 2%, 3%, and 4% SA, there was a pattern of “preflowering accumulation → flowering peak → slight postflowering decline, “ with 2% SA yielding the highest absorbance during flowering. Differences in postflowering energy storage were evident with 5% SA, where the postflowering absorbance was significantly greater than that before flowering (only a 10.4% decrease). With 6% and 7% SA, the postflowering absorbance was lower than that before flowering (decreases of 44.2% and 50.4%, respectively). With 2% SA, there was sustained high expression from preflowering to flowering, whereas 5% SA resulted in the smallest fluctuation across all three periods (CV = 9.7%). Data related to the dynamic changes in starch content are shown in **Supplementary Tables A5**, **A6**.

#### 3.4.4 Dynamic changes in protein content

**Figure 5:**
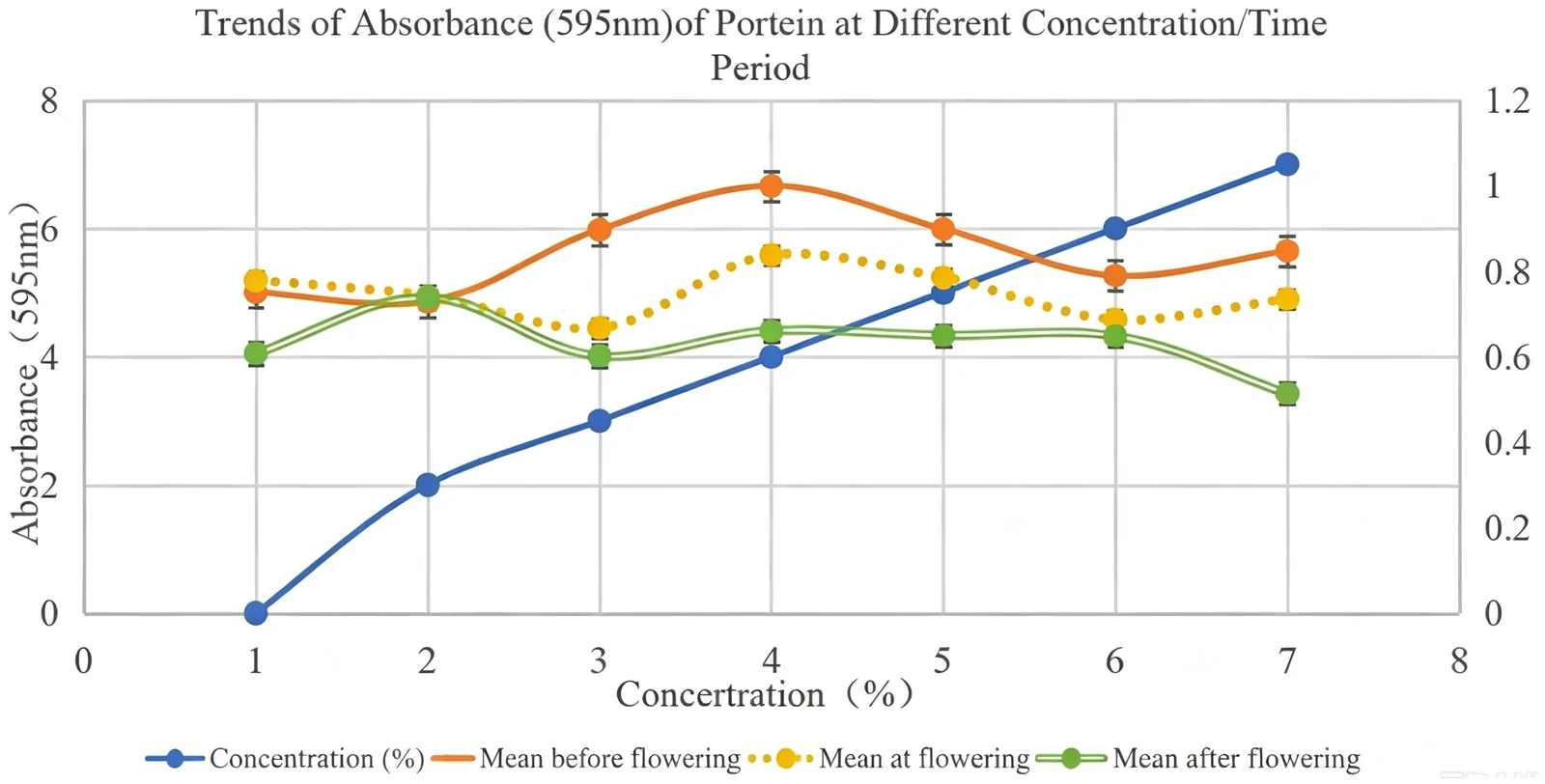
Absorbance (595 nm) of protein as a function of different concentrations/time periods.

A consistent downwards trend in protein content across all groups from flowering to postflowering is shown in **Figure 5**, indicating protein consumption or transfer during postflowering stages (e.g., fruit set and senescence). Protein content in the CK group slightly increased from preflowering to flowering, whereas in the treated groups (3%, 4%, and 5% SA), protein content generally decreased. In the 3% SA group, protein content significantly decreased, with moderate decreases observed in the 4% and 5% SA groups. The optimization potential of 4% SA was evident through significant protein accumulation before flowering, providing ample enzymes and structural proteins for flowering, although protein content declined rapidly postflowering (-21.15%). In the 5% SA group, there was balanced regulation, with the smallest overall protein reduction (-12.68% to -17.19%). The 3% SA group showed limitations, with greater protein depletion during flowering (-25.67%). Higher concentrations (3% SA/4% SA) significantly influence preflowering protein accumulation or consumption, whereas lower concentrations (5% SA) prove more suitable postflowering. The data on the dynamic changes in protein content are shown in **Supplementary Tables A7**, **A8**.

Exogenous salicylic acid (SA) exerts concentration-dependent remodeling of carbon partitioning and source–sink balance in Brassicaceae crops, coordinating the metabolism of soluble sugars, sucrose, starch and soluble proteins to reallocate photosynthates between source leaves, transport phloem and reproductive sink tissues (Hayat et al., 2003; Martínez C et al., 2004).

### 3.5 Response surface model construction and validation

Based on the results of single-factor experiments, the Box-Behnken design was adopted, and Design-Expert 13 software was used for regression fitting analysis of protein content (see **Table 10**). Consequently, model parameters and analysis-of-variance (ANOVA) results were obtained (see Tables 3.27, 3.28, 3.29), together with the multiple quadratic regression equations derived from the general response surface formula presented in Equation 3: Y_1_** = **47.72−0.0700A−0.7433B−0.1400AB−5.09A^2^−3.22B^2^.By analyzing the absolute values and positive-negative signs in the above quadratic polynomial equations, the magnitude and direction of effects of various factors on the response value Y (protein content) can be revealed. First, the negative coefficients of A^2^ and B^2^ indicate that the quadratic equation describes a downward-opening paraboloid. Within a certain range, the response value Y increases first and then decreases with rising levels of these factors (concentration and treatment time), suggesting the existence of an optimal condition combination for maximizing protein content. Second, the relative effect magnitude of each factor on response Y can be judged by comparing the absolute values of the linear coefficients of A and B. The factor influence order is: B (treatment time) > A (concentration). Optimizing the optimal combination of these factors can markedly improve protein content.Y^2^** = **56.60 + 0.9600A-0.6967B+1.70AB-6.09A^2^-3.07B^2^ The factor influence order is: A (concentration) > B (treatment time). Optimizing the optimal combination of these factors can markedly improve sugar content.Y_3_** = **67.78 + 1.17A+1.69B-0.9750AB-7.52A^2^-4.40B^2^.The factor influence order is: B (treatment time) > A (concentration). Optimizing the optimal combination of these factors can markedly improve starch content. ANOVA results for the above models are presented in **Tables 11**–**13**.

**Table 10:** Analysis of response surface methodology experimental results.

No.	A: concentration (%)	B: periods (d)	Protein (mg)	Carbohydrate (mg)	Starch (mg)
1	3	15	40.43	48.96	51.48
2	7	15	40.15	47.57	56.16
3	3	35	39.52	44.08	57.72
4	7	35	38.68	49.48	58.5
5	3	25	41.73	49.48	59.28
6	7	25	42.43	51.23	60.84
7	5	15	44.99	53.98	62.4
8	5	35	42.91	52.77	63.96
9	5	25	47.41	55.21	68.64
10	5	25	48.32	55.68	67.08
11	5	25	47.72	57.96	67.08
12	5	25	47.72	57.77	67.86
13	5	25	48.53	56.72	68.64

**Table 11:** ANOVA of regression equation terms (protein).

Variance source	SS	DF	MS	F number	P number	Significance
Model	160.61	5	32.12	65.36	< 0.0001	**
A-Concentration	0.0294	1	0.0294	0.0598	0.8138	
B-Periods	3.32	1	3.32	6.75	0.0356	*
AB	0.0784	1	0.0784	0.1595	0.7015	
A^2^	71.42	1	71.42	145.33	< 0.0001	**
B^2^	28.55	1	28.55	58.10	0.0001	**
Residual	3.44	7	0.4914			
Lack-of-Fit-Term	2.57	3	0.8566	3.94	0.1093	
Theoretical Error	0.8702	4	0.2176			
Total Deviation	164.05	12				
Std. Dev.	0.7010			R^2^	0.9790	
Mean	43.89			R^2^ Adj	0.9641	
C.V. %	1.60			R^2^ Pred	0.8692	
				Adeq Prec	19.4303	

*P<0.05, significant difference; **P<0.01, highly significantly different.

**Table 12:** ANOVA of regression equation terms (soluble sugars).

Variance source	SS	DF	MS	F number	P number	Significance
Model	216.32	5	43.26	48.99	< 0.0001	**
A-Concentration	5.53	1	5.53	6.26	0.0409	*
B-Periods	2.91	1	2.91	3.30	0.1122	
AB	11.53	1	11.53	13.05	0.0086	**
A^2^	102.46	1	102.46	116.03	< 0.0001	**
B^2^	26.04	1	26.04	29.49	0.0010	**
Residual	6.18	7	0.8831			
Lack-of-Fit-Term	0.1931	3	0.0644	0.0430	0.9865	
Theoretical Error	5.99	4	1.50			
Total Deviation	222.50	12				
Std. Dev.	0.9397			R^2^	0.9722	
Mean	52.38			R^2^ Adj	0.9524	
C.V. %	1.79			R^2^ Pred	0.9541	
				Adeq Prec	19.6043	

*P<0.05, significant difference; **P<0.01, highly significantly different.

**Table 13:** ANOVA of regression equation terms (starch).

Variance source	SS	DF	MS	F number	P number	Significance
Model	355.59	5	71.12	88.74	< 0.0001	**
A-Concentration	8.21	1	8.21	10.25	0.0150	*
B-Periods	17.14	1	17.14	21.38	0.0024	**
AB	3.80	1	3.80	4.74	0.0658	
A^2^	156.09	1	156.09	194.75	< 0.0001	**
B^2^	53.41	1	53.41	66.64	< 0.0001	**
Residual	5.61	7	0.8015			
Lack-of-Fit-Term	3.18	3	1.06	1.74	0.2967	
Theoretical Error	2.43	4	0.6084			
Total Deviation	361.20	12				
Std. Dev.	0.8952			R^2^	0.9845	
Mean	62.28			R^2^ Adj	0.9734	
C.V. %	1.44			R^2^ Pred	0.9028	
				Adeq Prec	25.8965	

*P<0.05, significant difference; **P<0.01, highly significantly different.

The data in **Tables 11**–**13** demonstrate highly significant model fit, with protein, sugar, and starch contents strongly influenced by the selected factors. Both the primary term A and the interaction term AB yielded P values greater than 0.05, indicating that they have no significant effect on protein, sugar, or starch content. On the basis of the F values, multiple factors influence protein, sugar, and starch contents. With respect to protein and starch, factor B (time period) had the most significant effect, whereas factor A (concentration) had a weaker effect. With respect to sugars, factor A (concentration) had the most pronounced effect, whereas factor B (time period) had a weaker influence.

**Figure 6:**
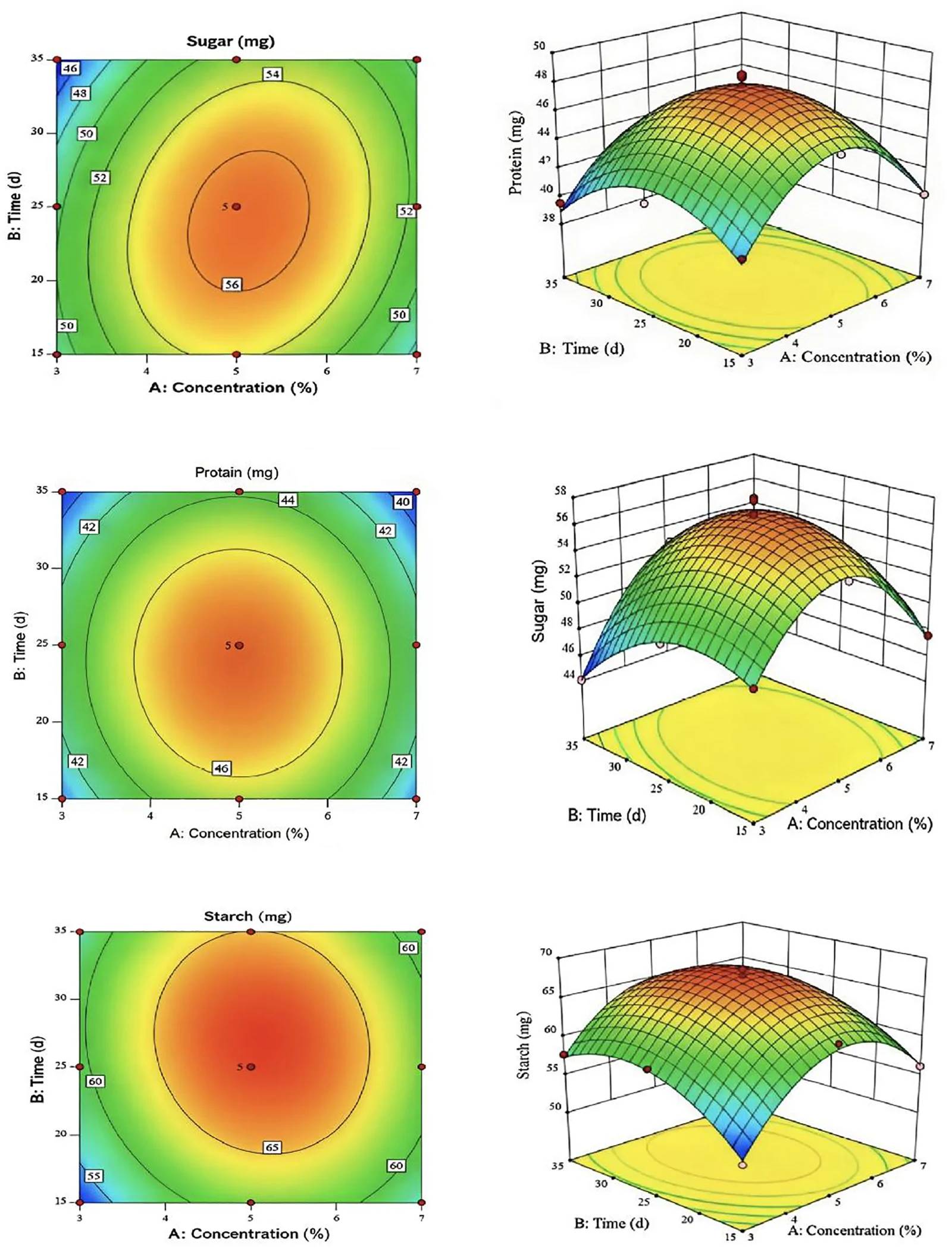
Response surface plot of effects of concentration and time period on proteins, soluble sugars and starch.

A regression model was constructed using regression equations, generating a three-dimensional graph and contour lines. As shown in **Figure 6**, with increasing concentration (A), the protein content first increased but then decreased; similarly, with increasing period (B), the protein content first increased but then decreased. The results of the variance analysis indicate that P(AB) > 0.05, indicating that the interaction between the two factors had a minor effect on the results. A contour plot revealed that protein levels were greater when the concentration (A) was between 4% and 6% and the period (B) was between 20 and 30 days.As the concentration (A) increased, the sugar content first increased but then decreased; similarly, as the period (B) increased, the sugar content first increased but then decreased. The variance analysis showed P(AB)< 0.05, indicating that the interaction between the two factors significantly influenced the results. A contour plot revealed that the sugar content was highest when the concentration (A) was between 4% and 6% and the period (B) was between 20 and 30 days. As the concentration (A) increased, the starch content first increased but then decreased; similarly, as the period (B) increased, the starch content tended to first increase but then decrease. The variance analysis results showed P(AB) > 0.05, indicating that the interaction between the two factors had a minor effect on the results. A contour plot revealed that the starch content was highest when the concentration (A) ranged from 4–6% and the incubation period (B) spanned 25**–**30 days. The response surface exhibited a downwards-opening shape, suggesting that as the factor levels increased, the protein, sugar, and starch contents gradually increased and peaked before they decreased. The slope of the response surface indicated that steeper slopes signified a more significant interaction effect between factors on protein content. The figure shows a steeper slope for sugar AB, indicating that the interaction between concentration and incubation period had a more pronounced effect on the sugar content, while its influence on protein and starch was relatively minor.

**Figure 7:**
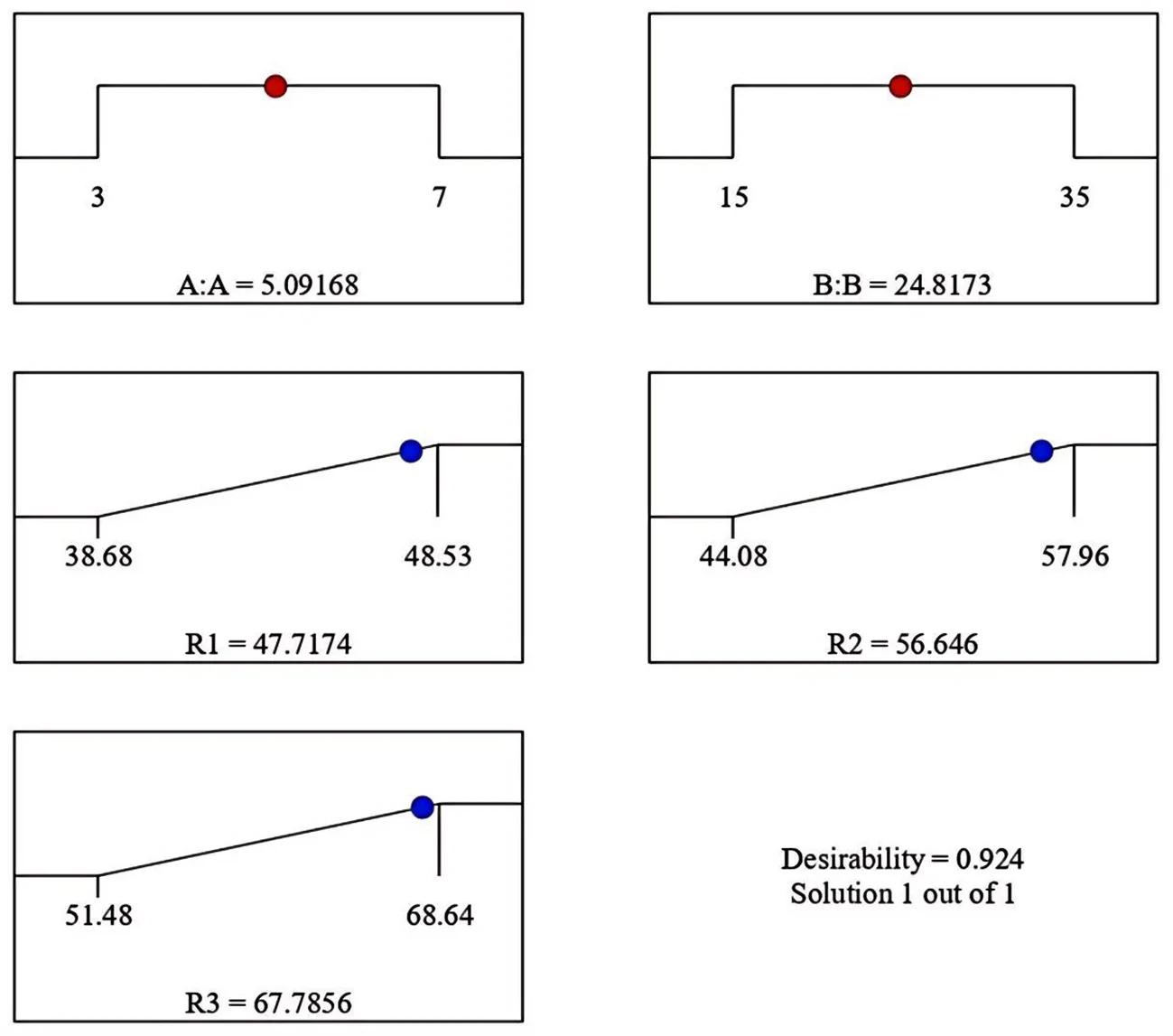
Overall model validation plot.

As shown in **Figure 7**, according to the optimal model, the optimal contents of protein, sugar and starch in Red radish *(Raphanus sativus L.*)can be achieved at a concentration of 5.09 % and a treatment time of 24.82d, with the predicted values of 47.72 mg, 56.65 mg and 67.79 mg, respectively. Considering the feasibility and convenience of practical operation, the optimal extraction conditions were adjusted as follows: concentration of 5.10 % and treatment time of 25 d. Verification experiments were carried out using this optimized process. The experimental results showed that the actual contents of protein, sugar and starch were 47.28 mg, 56.28 mg and 67.15 mg, respectively. The relative errors between experimental and predicted values were only 0.92 %, 0.65 % and 0.94 %, indicating that the experimental results were highly consistent with the predicted values and fully demonstrating the accuracy and reliability of the established model.

### 3.6 Regulatory effects of SA treatment on target flowering gene expression

**Figure 8:**
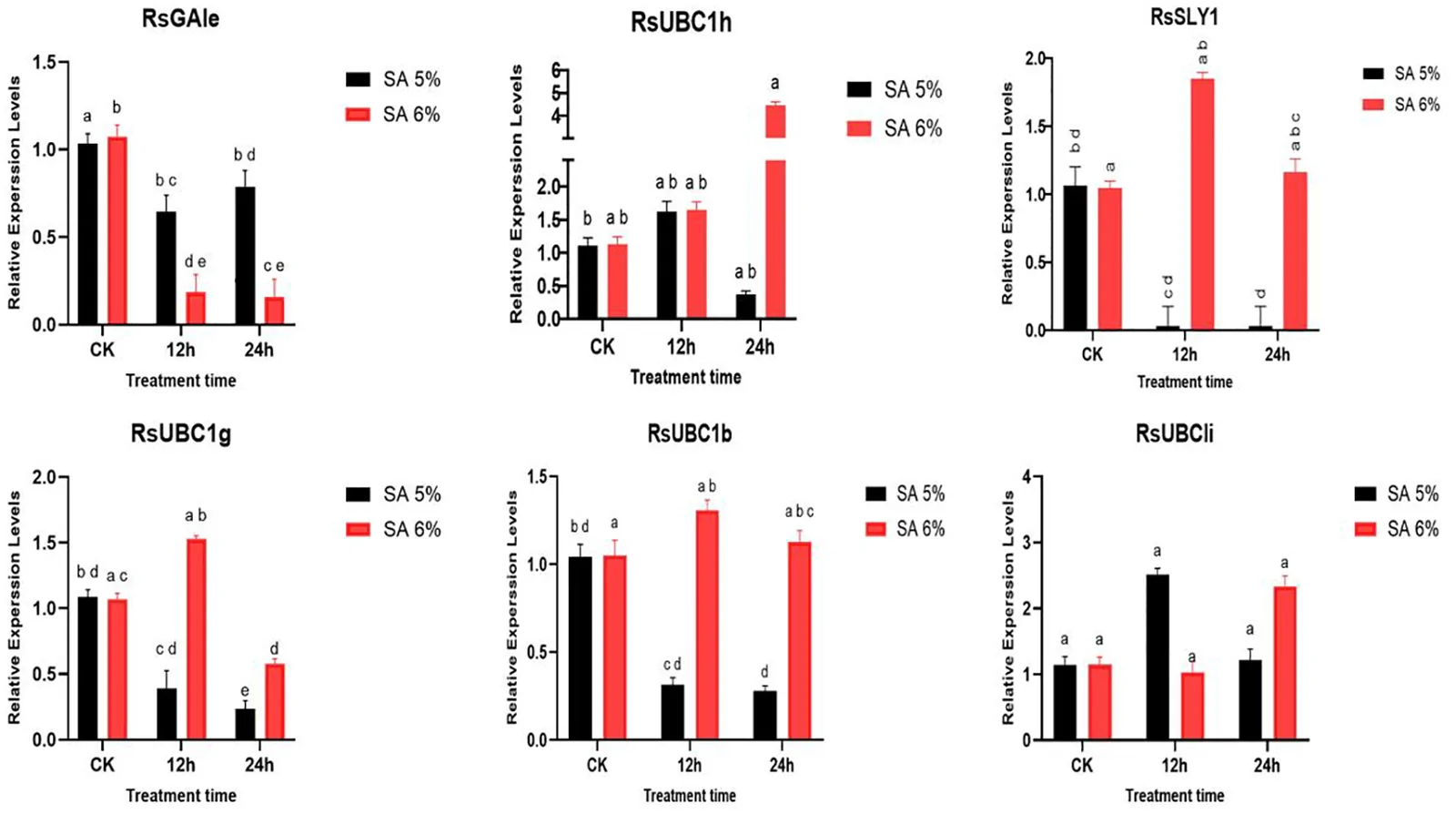
Relative expression of key flowering genes in *Raphan sativus L*. Different lowercase letters above bars indicate statistically significant differences at P < 0.05 according to one‑way ANOVA followed by Duncan’s multiple‑range test. Error bars represent the standard error of the mean (SE).

qRT–PCR was performed to detect the relative expression levels of six candidate genes (RsGAIe(DELLA), RsUBC1h/g/b/i(E2-UBC1)), photoperiod and flowering regulatory genes RsSLY1, In response to 5% and 6% SA treatments at different time points (CK, 0, 12, and 24 h). Statistical significance was analysed by two-way analysis of variance (Two-way ANOVA) at a significance level of P< 0.05, and the results are presented in **Figure 8**. Our results revealed that SA significantly up-regulated RsSLY1 and RsUBC1h/g/b, while repressing the transcription of the negative-regulator gene RsGAIe. By contrast, RsUBC1i showed no transcriptional response to SA.

**Figure 8** illustrates that SA promotes the accumulation of bioactive GA_3_ in leaves, and this regulatory effect was more pronounced under 6% SA treatment compared with 5% SA, in agreement with the data in **Table 4**. SA induces increased transcript levels of RsSLY1 and RsUBC1h/g/b/i, while down-regulation of RsGAIe alleviates this repressive effect, sustains normal IAA transport and homeostasis in leaf tissues, and facilitates CTK biosynthesis, consistent with the results in **Table 6**. Furthermore, 6% SA significantly suppressed RsGAIe transcription in leaves. Reduced RsGAIe transcript abundance, together with elevated expression of SLY1 and RsUBC1h/g, stimulated the accumulation of soluble sugars, starch, and proteins in leaf tissues, which aligns with the findings presented in **Figures 2**–**4**. These changes may interfere with the DELLA-SLY1 interaction (Chen et al., 2022; Hernández-García et al., 2020).

## 4 Discussion

### 4.1 Effects of SA concentration on endogenous hormone dynamics in the red radish

This study revealed a concentration-dependent regulatory pattern of SA on endogenous GA_3_, IAA, and CTK homeostasis (Zhao, 2023; Cheng et al., 2025), which governs flower-bud differentiation and flowering in red radish. Consistent with previous Brassicaceae studies (Zou et al., 2020; Li et al., 2024), moderate SA (2–5%) elevated GA_3_ levels to promote vegetative biomass accumulation for reproductive transition, while high-concentration SA (7%) suppressed GA_3_ activity and arrested growth (Zwack & Rashotte, 2013). Moderate SA also increased CTK content to delay leaf senescence and sustain photosynthetic supply for flowering (Zwack & Rashotte, 2013). SA-induced apical IAA reduction may redirect auxin distribution to alter bud development, though this hypothesis requires further validation. Distinct from Arabidopsis, radish exhibits unique organ-specific hormone responses. This study only detected hormonal changes; future molecular assays are needed to clarify the underlying regulatory mechanism.

### 4.2 Regulatory effects of SA on sugar metabolism and flowering

SA regulates plant floral transition through carbon metabolic reprogramming, which is conserved in Brassicaceae species (Martínez et al., 2004). Consistent with previous crop studies, moderate 5% SA increased pre-flowering soluble sugar and fructose levels to provide carbon substrates for flower-bud differentiation, accompanied by dynamic sugar consumption during floral development via the classic SnRK1-TOR sugar signaling pathway (Baena-González & Sheen, 2008). Differing from Arabidopsis, radish exhibited obvious post-flowering sugar and starch recovery under optimal SA treatment, reflecting a unique carbon allocation strategy balancing reproductive growth and tuberous root nutrition. In contrast, high SA (6%–7%) caused irreversible carbon depletion and disrupted floral development (Li et al., 2024). This study only detected metabolic phenotypes; the potential regulation of SPS and starch synthesis remains unvalidated and requires further molecular verification.

### 4.3 Optimizing the effects of SA on pollen characteristics and pollination efficiency

This study identified a typical dual concentration-dependent effect of SA on radish pollen development, which complements and refines the SA-mediated reproductive regulatory mechanism in Brassicaceae. Furthermore, pollen viability is closely linked to sugar metabolism: in the 5% SA group, higher fructose levels were maintained during flowering, providing sufficient energy for pollen germination (Ying et al., 2022). Notably, all pollen assessments in this study relied on I_2_-KI staining, a rapid qualitative method with inherent limitations. This technique only reflects starch accumulation and may produce false-positive results, failing to distinguish weakly viable pollen compared with authoritative *in vitro* germination or FDA staining. Therefore, the conclusion regarding SA-regulated pollen performance remains phenotypically correlative; future multi-method validation and molecular detection of tapetum development and ROS signaling are required to clarify the underlying mechanism.

### 4.4 Gene-physiological trait association analysis based on qPCR expression profiles

SA exerted prominent concentration- and time-dependent biological effects. Moderate SA triggered coordinated gene expression to sustain phytohormone balance, promote floral transition and boost carbon-nitrogen metabolite accumulation. Prolonged exposure to high-concentration SA, however, resulted in excessive activation of the ubiquitin system and severe circadian disorder, leading to physiological and metabolic imbalance. These molecular observations provide mechanistic support for the quadratic (rise-to-fall) responses of protein, soluble sugar and starch contents against These molecular changes provide credible genetic evidence for the quadratic rising-then-fall trend of physiological indices observed in the response surface model of this study.Nevertheless, gene expression was only assayed under the limited experimental conditions of CK, 5% SA and 6% SA, with sampling time-points set at 12 h and 24 h in the present study. Accordingly, there exist certain limitations, and further validation with expanded SA-concentration gradients and broader sampling-time ranges is needed.

### 4.5 Research limitations and future directions

Although this study elucidated the regulatory mechanism of SA on flowering in the red radish, Preliminary screening was performed across the concentration range of 1%–8%. After comprehensive evaluation, the range of 2%–7% was adopted for the formal experimental design, while potential effects at lower concentrations (e.g., 0.5%–1%) were not investigated. Second, the physiological measurements focused on sugars and hormones; therefore, molecular validation of key enzyme activities (e.g., SPS and invertase) and gene expression is lacking. Third, field trial conditions (e.g., temperature, humidity, and soil fertility) did not fully replicate actual production environments, potentially limiting the generalizability of the results. Future research should broaden the SA concentration range, integrate transcriptomic or metabolomic analyses to elucidate molecular mechanisms, and validate optimal concentration stability through large-scale field trials. Additionally, the synergistic effects between SA and other plant growth regulators (e.g., gibberellins and ethylene) warrant further investigation to develop more efficient techniques for regulating flowering.

## 5 Conclusions

Based on the results of single-factor experiments, the response surface methodology based on the established optimal model revealed that the treatment of red radish with 5.09% SA for 24.82 days contributed to the optimal accumulation of protein, soluble sugar, and starch, with predicted values of 47.72 mg, 56.65 mg, and 67.79 mg, respectively. To improve the practical operability of the treatment protocol in actual experiments, the optimized SA treatment conditions were appropriately adjusted to an SA concentration of 5.10% and a treatment duration of 25 days. Subsequent verification experiments adopting the adjusted optimal conditions obtained actual contents of 47.28 mg for protein, 56.28 mg for sugar, and 67.15 mg for starch. The relative errors between the experimental measured values and model predicted values were only 0.92%, 0.65%, and 0.94%, respectively. These results demonstrate a high consistency between experimental data and model simulation results, verifying the good fitting effect and practical applicability of the established response surface model under the current experimental conditions. Nevertheless, the present study only explored the regulatory effects of exogenous SA treatment on nutrient accumulation in red radish under laboratory controlled conditions. Given the complexity of field environmental conditions and the genetic diversity of radish genotypes, the universal applicability of the optimized SA treatment strategy still needs to be further validated via field trials and multi-genotype experiments. Follow-up studies are required to clarify the broader regulatory mechanism of SA on radish nutrient metabolism and provide more reliable theoretical and technical support for practical cultivation applications.

## Data Availability

The datasets presented in this study can be found in online repositories. The names of the repository/repositories and accession number(s) can be found in the article/**Supplementary Material**.
