# Supplementary material for: Salicylic acid exerts effects on regulating flower bud differentiation and nutrient contents in red radish
Source: Front Plant Sci. 2026 Sep 1;17:1914554. doi: 10.3389/fpls.2026.1914554 (PMC13574949; doi:10.3389/fpls.2026.1914554)
Supplement: Supplementary file 1 [file Table1.docx]

**Table A1.** Results of Two-way Analysis of Variance for Soluble Sugar Absorbance

| **Source of Variation** | **SS** | **df** | **MS** | **F** | **P** | **Partial Eta Squared**  **(η^2^P)** |
| --- | --- | --- | --- | --- | --- | --- |
| **Intercept** | 7.9 | 1 | 7.9 | 763.722 | 0.000** | 0.955 |
| **Time** | 0.469 | 2 | 0.235 | 22.686 | 0.000** | 0.558 |
| **Treatment** | 0.144 | 5 | 0.029 | 2.783 | 0.032* | 0.279 |
| **Time*Treatment** | 0.21 | 10 | 0.021 | 2.029 | 0.059 | 0.36 |
| **Residual** | 0.372 | 36 | 0.01 |  |  |  |

Note：R2 = 0.689，* p<0.05 ** p<0.01

**Table** A2. Concentration-Time Interaction

| **Concentration（%）** | **Rate of Change:**  **Preflowering**  **→Flowering** | **Rate of Change:**  **Flowering**  **→Postflowering** | **Physiological Significance** |
| --- | --- | --- | --- |
| **CK** | 136% | 6% | basal metabolism and no regulatory advantage |
| **2** | -36% | -45% | efficient accumulation before flowering, excessive consumption after flowering |
| **5** | -62% | 62% | optimal balance: sufficient energy supply during flowering and timely resupply after flowering |
| **7** | -45% | 35% | Moderate effect with weak stability |

**Table A3.** Results of Two-way Analysis of Variance for Sucrose Absorbance

| **Source of Variation** | **SS** | **df** | **MS** | **F** | **P** | **Partial Eta Squared**  **(η2P)** |
| --- | --- | --- | --- | --- | --- | --- |
| **Intercept** | 0.073 | 1 | 0.073 | 127.767 | 0.000** | 0.78 |
| **Time** | 0.002 | 5 | 0 | 0.876 | 0.507 | 0.108 |
| **Treatment** | 0.001 | 2 | 0.001 | 0.952 | 0.395 | 0.05 |
| **Time*Treatment** | 0.009 | 10 | 0.001 | 1.662 | 0.128 | 0.316 |
| **Residual** | 0.02 | 36 | 0.001 |  |  |  |

Note: R2 = 0.739，* p<0.05 ** p<0.01

**Table A4.** Comparison of Key Concentration of Sucrose

| **Concentration（%）** | **Rate of Change:**  **Preflowering**  **→Flowering** | **Rate of Change:**  **Flowering**  **→Postflowering** | **Physiological Significance** |
| --- | --- | --- | --- |
| **CK** | 56% | 6% | natural metabolism and no regulatory advantage |
| **3** | -66% | 61% | efficient energy supply and maintains stable nutrient reserves after flowering |
| **4** | 3% | 76% | Severe energy depletion during flowering with weak recovery capacity |
| **5** | 92% | 68% | Large fluctuations and uncertain reallocation capacity |

**Table A5.** Results of Two-way Analysis of Variance for Starch Absorbance

| **Source of Variation** | **SS** | **df** | **MS** | **F** | **P** | **Partial Eta Squared**  **(η2P)** |
| --- | --- | --- | --- | --- | --- | --- |
| **Intercept** | 3.247 | 1 | 3.247 | 817.554 | 0.000** | 0.958 |
| **Time** | 0.047 | 5 | 0.009 | 2.359 | 0.06 | 0.247 |
| **Treatment** | 0.035 | 2 | 0.018 | 4.408 | 0.019* | 0.197 |
| **Time*Treatment** | 0.053 | 10 | 0.005 | 1.347 | 0.244 | 0.272 |
| **Residual** | 0.143 | 36 | 0.004 |  |  |  |

**Table A6.** Comparison of Key Concentration of Starch

| **Concentration（%）** | **Rate of Change:**  **Preflowering**  **→Flowering** | **Rate of Change:**  **Flowering**  **→Postflowering** | **Physiological Significance** |
| --- | --- | --- | --- |
| **CK** | 33.6% | 0% | flowering accumulation and stable supply of development |
| **2** | 22.9% | -6.8% | flowering reserve accumunitions, moderate postflowering consumption |
| **3** | -14.7% | 12.7% | flowering consumption, reserve recovery postflowering |
| **5** | -21.4% | 14.1% | flowering translocation, reaccumulation after flowering |

**Table A7.** Results of Two-way Analysis of Variance for Protein Absorbance

| **Source of Variation** | **SS** | **df** | **MS** | **F** | **P** | **Partial Eta Squared**  **(η^2^P)** |
| --- | --- | --- | --- | --- | --- | --- |
| **Intercept** | 30.065 | 1 | 30.065 | 5468.884 | 0.000** | 0.993 |
| **Time** | 0.114 | 5 | 0.023 | 4.151 | 0.004** | 0.366 |
| **Treatment** | 0.454 | 2 | 0.227 | 41.317 | 0.000** | 0.697 |
| **Time*Treatment** | 0.159 | 10 | 0.016 | 2.889 | 0.009** | 0.445 |
| **Residual** | 0.198 | 36 | 0.005 |  |  |  |

Note: R2 = 0.739，* p<0.05 ** p<0.01

**Table A8.** Comparison of Key Concentration of Protein

| **Concentration（%）** | **Rate of Change:**  **Preflowering**  **→Flowering** | **Rate of Change:**  **Flowering**  **→Postflowering** | **Physiological Significance** |
| --- | --- | --- | --- |
| **CK** | +3.55% | -21.81% | initial enzyme activity requirement,reproductive costTranslocation |
| **3** | -25.67% | -9.66% | high concentration consumption inhibit synthesis postflowering |
| **4** | -16.30% | -21.15% | preferential allocation and dominant nautral senescence |
| **5** | -12.68% | -17.19% | maintaining metabolic support,transition maturity stage |
